# Heat and Moisture Exchangers and Humidification Efficacy in Pediatrics: Effects of Ventilator Settings and ETT Leakage

**DOI:** 10.1155/2012/585280

**Published:** 2012-08-01

**Authors:** Alan de Klerk, Antonio M. Esquinas

**Affiliations:** ^1^Department of Neonatology/BirthCare Center, Florida Hospital Memorial Medical Center, 301 Memorial Medical Parkway, Daytona Beach, FL 32114, USA; ^2^Intensive Care Unit, Hospital Morales Meseguer, Avenida Marques de Los Velez s/n, 30008 Murcia, Spain

Heat and moisture exchangers (HMEs) have been increasingly used for humidification during mechanical ventilation in pediatric patients [1]. However, efficacy of HMEs in the pediatric population has not yet been fully evaluated and there is limited information in the literature regarding the effects of ventilator settings and endotracheal tube (ETT) leakage on the humidification performance of HMEs.

Chikata et al. [2] tested ten pediatric HMEs in a model lung, measuring performance under different respiratory rates, tidal volumes TVs (achieved with different pressure control levels), and leakage conditions. They found that eight of the ten HMEs maintained absolute humidity (AH) at more than 30 mg/L, the minimum level recommended by the American Association for Respiratory Care (AARC). With a small leak, AH decreased below 30 mg/L (26.6 to 29.5 mg/L), decreasing further (19.7 to 27.3 mg/L) with a larger leak. Pressure control (tidal volume) level did not statistically significantly affect AH values, although there was a trend to decreased AH at higher tidal volumes. In three of the HMEs, increased respiratory rate resulted in a significantly higher AH. In six of the other seven HMEs, there was a nonstatistically significant trend to higher AH with increased respiratory rate, with one HME showing a nonsignificant trend to lower AH.

We think this is a well-designed and conducted study and provides valuable information regarding the use of HMEs in pediatric patients under different conditions. The effect of ETT leakage on AH is nicely demonstrated and is consistent with previously published data on mechanically ventilated adults.

However, we had some questions regarding the study's other findings and their possible explanation as follows

1. the study found that 8 out of 10 pediatric HMEs maintained AH at more than 30 mg/L, while they quote the Luchetti et al. [3] study they reference as showing “AH of less than 28 mg/L in all HMEs used with pediatric patients” despite using similar one-way valve methodology in their circuit to prevent mixing of inspired and expired gases? This finding is even more puzzling given that one of the two HMEs used in the Luchetti study, the Hygroboy (Covidien [formerly Tyco Healthcare], Mirandola, Italy), in the present study was found to produce the highest AH (32.4 mg/L) of the ten HMEs tested. How is this difference in findings between the 2 studies of the same HME explained?
2. the finding that increased respiratory rates resulted in a trend to higher AH that reached significance in 3 of the tested humidifiers is interesting. Previous studies of HMEs in adults and children have found lower AH associated with higher tidal volumes [4, 5] and minute volumes (MVs) [1, 6, 7]. It would seem more logical that an increasing respiratory rate presumably would result in a higher MV, with resultant lower AH, but Chikata et al. found the opposite. The authors did not comment on this in their paper, and we wonder if they have a plausible explanation for this;
3. finally, the authors speculate that the finding of a linear relationship between increasing dead space and increasing AH might possibly be related to improved humidification in those HMEs with larger internal volume and dead space. Since it is generally recommended to minimize dead space with HMEs, in particular in pediatric use, would the authors care to comment on striking a balance between maximizing humidification capacity and minimizing dead space?

## Abbreviations

AH: Absolute humidity
HMEs: Heat and moisture exchangers
TV: Tidal volume.
